# Temperature-Induced Irreversible Structural Transition in Fe_1.1_Mn_1.9_O_4_ Nanoparticles Synthesized by Combustion Method

**DOI:** 10.3390/nano13071273

**Published:** 2023-04-04

**Authors:** Aleksandr A. Spivakov, Chun-Rong Lin, Ying-Zhen Chen, Li-Huai Huang

**Affiliations:** Department of Applied Physics, National Pingtung University, No. 4-18 Minsheng Rd., Pingtung City 90003, Taiwan

**Keywords:** Fe_1.1_Mn_1.9_O_4_ nanoparticles, spinel ferrites, structural transition, high-temperature magnetic measurements, combustion method

## Abstract

Fe_1.1_Mn_1.9_O_4_ nanoparticles were successfully synthesized using a combustion method. The influence of the heating temperature on the evolution of the structural and magnetic properties has been studied using various methods. The structural analysis results revealed that as-synthesized nanoparticles have a tetragonal structure with an average size of ~24 nm. The magnetic measurements of the sample showed its ferrimagnetic nature at room temperature with hysteresis at low fields. Temperature-dependent magnetization measurements allowed for the conclusion that the Curie temperature for Fe_1.1_Mn_1.9_O_4_ nanoparticles was ~465 °C. After high-temperature magnetic measurements, during which the samples were heated to various maximum heating temperatures (T_max.heat._) in the range from 500 to 900 °C, it was found that the structure of the samples after cooling to room temperature depended on the heating temperature. Herewith, when the heating temperature was 600 < T_max.heat._ < 700 °C, an irreversible structural phase transition occurred, and the cooled samples retained a high-temperature cubic structure. The results of the magnetic analysis showed that the samples, following high-temperature magnetic measurements, demonstrated ferrimagnetic behavior.

## 1. Introduction

Due to their unique properties, spinel ferrites have attracted increasing interest in both fundamental scientific and practical application points of view [1,2,3]. Since magnetite (Fe_3_O_4_, the structure can be written in the form (Fe^3+^)_A_[Fe^2+^Fe^3+^]_B_O_4_, where () and [] are denoting tetrahedral A and octahedral B sublattices, respectively), nanoparticles have found practical applications in a wide variety of fields [4,5,6,7,8]; their study is of particular interest to researchers. Of additional interest is the possibility of improving the properties of magnetite by doping with metal ions, such as Mn, Co, Ni, Cu, etc. [9,10,11]. Among various cation-substituted ferrites, the Mn_x_Fe_3−x_O_4_ system has long attracted the attention of researchers due to the possibility of changing the physical properties by changing the concentration of manganese, which increases the possible applications of the system [12,13,14,15,16,17,18,19,20]. Various methods of synthesizing Mn_x_Fe_3−x_O_4_ nanoparticles have been described in the literature, such as sol-gel, hydrothermal, combustion, co-precipitation, mechanochemical approaches, and so on [21,22,23,24,25]. Some conventional techniques (such as co-precipitation and mechanochemical approaches) have a long sintering time or the obtained particles have a wide distribution of particle sizes [22,25]. The sol-gel method allows for the synthesis of nanoparticles with good stoichiometry and the control of particle size distribution, but this method requires heat treatment at high temperatures [23]. Among various synthesis techniques, the combustion method has attracted interest due to the high purity and homogeneity of the obtained nanoparticles, and in addition, it does not require sophistical equipment.

However, most studies of the Mn_x_Fe_3−x_O_4_ system have focused on the iron-reach region (*x* ≤ 1), while data on the study [26,27,28,29] and practical applications [30,31,32] of samples from the manganese-rich region (*x* ~ 2) are limited in the literature. It was found that, at *x* < 1.9, the system crystallized in a cubic structure, and at *x* > 1.9, it crystallized in a tetragonal structure (for bulk and single crystal samples) [33]. Brabers [26] showed that, in the single-crystal sample (*x* = 1.9), with an increase in temperature to 250–350 °C, a change occurred in the cation distribution, which depends on the composition of the sample [34]. This redistribution was stabilized through the tetragonal distortion of the lattice due to the Jahn–Teller effect [33,35]. With a subsequent increase in *x*, the concentration of octahedral Mn^3+^ ions became critical, and the transition occurred even at room temperature. It is known that a decrease in the size of particles to the nanoscale significantly affects their physicochemical properties; however, studies of nanoparticles of the Mn_x_Fe_3−x_O_4_ system at *x* ~ 2 are limited. Therefore, the study of such nanoparticles and the influence of various parameters on their structural and physical properties is an important task contributing to the progress of their practical application.

In light of the lack of literature on the study of Mn_x_Fe_3−x_O_4_ nanoparticles from the manganese-rich region and the fact that the transition to the nanoscale region can have a significant effect on the properties of the samples, the purpose of this study was to synthesize Fe_1.1_Mn_1.9_O_4_ nanoparticles through the combustion method and study their physicochemical properties. The influence of the maximum heating temperature on the structural changes and magnetic properties of the obtained nanoparticles was also studied and discussed. The obtained results can be used for further study of the Mn-rich region of the Mn_x_Fe_3−x_O_4_ system in the nanoscale region and the development of the practical applications of such nanoparticles.

## 2. Materials and Methods

### 2.1. Synthesis of Fe_1.1_Mn_1.9_O_4_ Nanoparticles

In the present work, Fe_1.1_Mn_1.9_O_4_ nanoparticles were synthesized through the combustion method using standard complex precursors. In this process, 2.9 g of manganese (II) nitrate tetrahydrate (Mn(NO_3_)_2_·4H_2_O) and 8 g of iron (III) nitrate nonahydrate (Fe(NO_3_)_3_·9H_2_O) were dissolved in a solution of citric acid, glycine, and deionized water. The obtained solution was then heated to 130 °C under magnetic stirring to form the gel. When the solution became homogeneous, the temperature was raised to 200 °C for 10 min. The gel ignites spontaneously and forms an aggregate of loose powders. The resulting product was placed in an oven heated to 200 °C and annealed at T_A_ = 1100 °C for 5 h; immediately after annealing, the sample was quenched in air.

The resulting nanoparticles were divided into samples and named S1—as-synthesized sample; S2–S7 samples, which were subjected to heating at temperatures of 500, 550, 600, 700, 800, and 900 °C, respectively, during magnetic measurements.

### 2.2. Characterization

The structural properties of the samples were studied using X-ray powder diffraction (XRD) using a Shimadzu XRD-6000 diffractometer (Cu Ka radiation, 40 kV, 30 mA, λ = 1.5406 Å). The morphology of the nanoparticles was characterized using the JEOL JEM-1230 transmission electron microscope operated at an accelerating voltage of 120 kV. Magnetic properties were studied using a vibrating sample magnetometer Lakeshore 7400 series VSM at temperatures between 30 °C and a maximum measurement temperature of 900 °C in an applied field of H = ±18 kilo-oersteds (kOe).

## 3. Results and Discussions

### 3.1. Structural and Magnetic Properties of the As-Synthesized Sample

**Figure 1:**
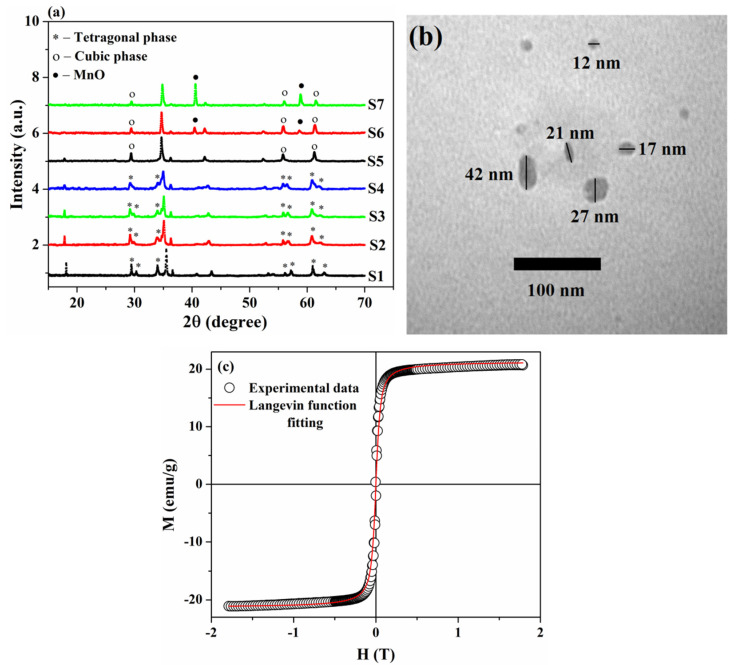
(**a**) XRD pattern of the as–synthesized sample, (**b**) TEM image of the as-synthesized Fe_1.1_Mn_1.9_O_4_ nanoparticles, and (**c**) magnetic hysteresis loop of S1 sample fitted by the Langevin function.

The XRD pattern of the as-synthesized sample is shown in Figure 1a. The diffraction peaks of the sample corresponded to the planes indexed ((101), (112), (200), (103), (211), (202), (004), (220), (312), (105), (303), (321), (224), and (400)), and they were consistent with a tetragonal spinel structure of FeMn_2_O_4_ [28]. However, as was shown by Brabers [26], a single crystal Fe_1.1_Mn_1.9_O_4_ sample still has a cubic structure at room temperature. The formation of a tetragonal structure can be related to the fact that a decrease in the particle size contributes to the redistribution of cations between the tetrahedral and octahedral sites in a spinel structure [36,37,38]; as a result of this, the cubic–tetragonal transition in this sample could occur at room temperature.

The values of the lattice parameters, calculated from the XRD data, were *a* = *b* = 5.95 Å, *c* = 8.86 Å (or, in pseudocubic notation $a^{\prime }=\sqrt{2}a=8.41$, $c^{\prime }=c=8.86$), and these values were close to those calculated in [39] and the experimental [26,28] values of FeMn_2_O_4_. The average crystallite size of the sample was calculated following Scherrer’s Equation (1), and the calculated value was ~20.5 nm.

(1) $$D=\frac{0.89\lambda }{\beta \cos\theta }$$

where *λ* is the radiation wavelength (0.15406 nm for Cu Kα) and *β* is the line broadening of a diffraction peak at angle *θ*.

The TEM image of the as-synthesized sample is shown in Figure 1b and demonstrates the sizes and morphology of the sample. It can be seen that nanoparticles obtained through the combustion method were well separated and almost uniform in size, with a spherical or quasi-spherical shape. The particle size of the as-synthesized sample oscillated between 11 and 45 nm, with an average size of ~24 nm, and this value agreed well with the result obtained using XRD analysis.

The room-temperature magnetic hysteresis loop of the S1 sample is shown in Figure 1c. The hysteresis loop demonstrates the ferrimagnetic behavior of the sample with low coercivity and remanence magnetization, which is a characteristic of soft magnetic materials. The saturation magnetization, coercivity, and remanent magnetization values obtained from the M–H curve were 20.9 emu/g, 38.8 Oe, and 2.3 emu/g, respectively. Since the value of remanent magnetization was low, the hysteresis loop could be fitted using the Langevin function, which is given as:

(2) $$M=M_{S}\left[coth\left(\mu H/k_{B}T\right)-k_{B}T/\mu H\right]$$

where *k_B_* is the Boltzmann constant, *T* is the absolute temperature, *M_S_* is the saturation magnetization, *μ* is the mean magnetic moment, and *H* is the applied magnetic field. From Figure 1c, it can be observed that the M–H curve fits well enough with the Langevin function, and the value of saturation magnetization, obtained from the fitting, was 21.44 emu/g which was close to the experimental value.

### 3.2. Temperature-Dependent Magnetization Measurements

To further study the stability of the obtained nanoparticles and the effect of T_max.heat._ on their structural and magnetic properties, we studied the dependence of magnetization on the temperature in an applied field of 1000 Oe. Herewith, the maximum heating temperature varied in the range of 500 ≤ T_max.heat._ ≤ 900 °C. 

**Figure 2:**
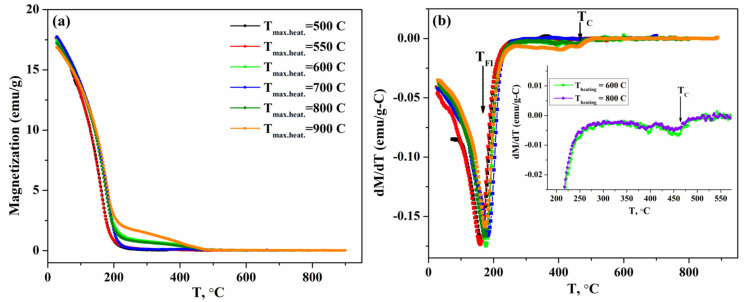
(**a**) Temperature–dependent magnetization of the Fe_1.1_Mn_1.9_O_4_ nanoparticles heated to 500 ≤ T_max.heat._ ≤ 900 °C; (**b**) derivative of M with respect to temperature. The inset in (**b**) shows dM/dT on an enlarged scale near T_C_ for the samples heated to 600 and 800 °C.

The temperature dependencies of the magnetization of the samples heated to 500 ≤ T_max.heat._ ≤ 900 °C are presented in Figure 2a, and Figure 2b shows the dependencies dM/dT—T for better identification of the transition temperatures. The transition at temperature T_FI_ ~ 179 °C (Figure 2b) was a magnetic transition, which corresponded to the ferrimagnetic magnetic ordering and has been observed in single-crystal FeMn_2_O_4_ samples [28]. Another poorly resolved anomaly (inset in Figure 2b) was observed at a temperature of around 465 °C.

R. Nepal and co-authors found that the Curie temperature for FeMn_2_O_4_ single crystals was ~322 °C. However, studies of MnFe_2_O_4_ nanoparticles have shown that a decrease in particle size leads to a significant increase in the Curie temperature [40,41] compared with bulk samples. Therefore, it can be assumed that this anomaly corresponds to the structural transition from a cubic structure at high to a tetragonal structure at low temperatures in Fe_1.1_Mn_1.9_O_4_ nanoparticles. Thus, during the high-temperature magnetic measurements, samples S2–S7 were heated above the Curie temperature and transformed to a cubic phase, after which they were cooled to room temperature for further study. 

### 3.3. Effect of High-Temperature Measurements on the Structural and Magnetic Properties

**Figure 3:**
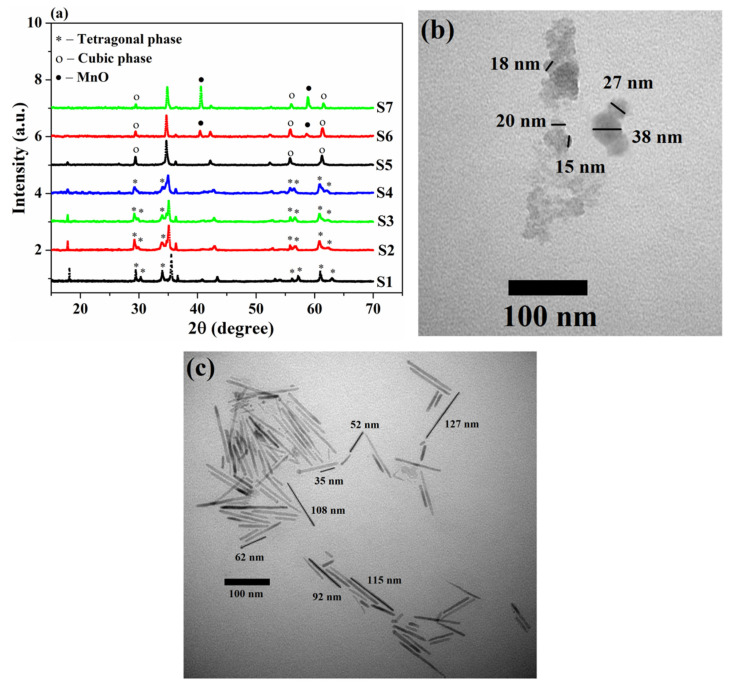
(**a**) XRD patterns of the as-synthesized sample and samples after heating to various temperatures; (**b**,**c**) TEM images of the samples heated during high-temperature magnetic measurements to 600 °C and 700 °C, respectively.

The samples after high-temperature magnetic measurements were again subjected to XRD analysis, and the obtained patterns are presented in Figure 3a. The average crystallite size of samples S2–S4 was calculated according to Equation (1), and the obtained values are provided in Table 1. The obtained results showed that the size of crystallites increased for the sample heated to 500 °C compared to the as-synthesized sample. However, a subsequent increase in T_max.heat_ to 550 and 600 °C led to a gradual decrease in the crystallite size. As will be discussed further, the subsequent increase in T_max.heat._ leads to a change in the shape of nanoparticles to needle-like; therefore, for samples S5–S7, the term ‘crystallite size’ is not appropriate since such nanoparticles require at least two geometric parameters to describe their shape. The results obtained demonstrate that when the heating temperature is less than 700 °C, the Fe_1.1_Mn_1.9_O_4_ nanoparticles retain their tetragonal structure after cooling. Nevertheless, it can be seen that with increasing heating temperature, the position of the most intense peak at 35.5° shifted toward smaller angles, and the well-separated peaks at 29.45° and 30.3°, 56.2° and 57.25°, 61° and 63° gradually merged. However, for the sample heated to T_max.heat._ = 700 °C, all peaks after their cooling were in good agreement with the cubic inverse spinel structure of magnetite (JCPDS 19-0629) [42] and did not contain features of the tetragonal phase or impurities. Thus, an increase in the heating temperature led to the tetragonal–cubic structural transition becoming irreversible and the cubic structure retaining even after cooling to room temperature. A further increase in T_heating_ led to peaks appearing in the XRD patterns corresponding to manganese (II) oxide (MnO) [43,44]. At the same time, with the increase in T_max.heat._ from 800 to 900 °C, the amount of MnO increased significantly. Thus, it can be concluded that, at a T_max.heat._ ≥ 800 °C, the degradation of the synthesized nanoparticles occurs.

Figure 3b,c shows the morphology of the nanoparticles after heating to 600 and 700 °C, respectively. It can be seen that particles heated to 600 °C still retained spherical and quasi-spherical shapes, as in the case of the S1 sample, with an average size of around 21 nm. However, in contrast to the as-synthesized sample with well-separated nanoparticles, in the sample heated to 600 °C, the nanoparticles demonstrated a tendency to agglomerate, forming elongated structures. The further increase in T_max.heat._ led to the nanoparticles, after heating, having a needle-like structure with lengths ranging between 30 and 130 nm (average length ~ 97 nm) and a thickness of about 6 nm. Thus, the heating of as-synthesized nanoparticles led not only to a change in the structure of the samples but also to a change in their morphology. 

**Figure 4:**
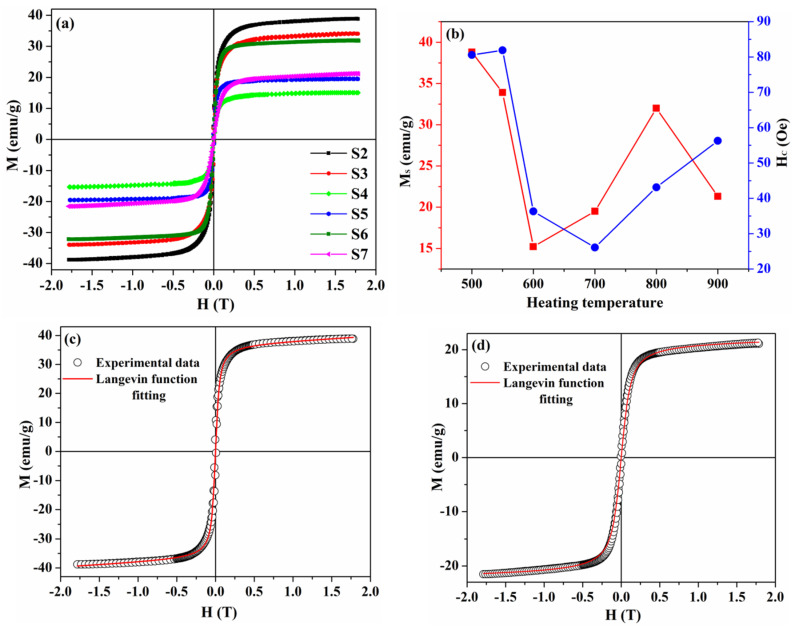
(**a**) Magnetic hysteresis loops of the samples, heated to various temperatures; (**b**) dependence of saturation magnetization and coercivity on heating temperature; (**c**,**d**) selected magnetic hysteresis loops fitted by Langevin function for samples S2 and S7, respectively.

The magnetic hysteresis loops of the S1–S7 samples measured at room temperature are shown in Figure 4a,b, which presents the hysteresis loops on an enlarged scale. 

The M–H curves of the samples, after high-temperature magnetic measurements, demonstrate ferrimagnetic behavior at room temperature with hysteresis at low fields and with the values of saturation magnetization, coercivity, and remanent magnetization listed in Table 1.

The results of magnetic measurements demonstrate that the saturation magnetization of sample S2 increased compared to sample S1, and for samples S3–S4, a decrease in saturation magnetization was observed, which is in good agreement with the change in the crystallite size discussed above. As follows from the TEM data, an increase in T_max.heat_ > 600 °C led to a change in the size and shape of the samples, which led to an increase in the saturation magnetization in samples S5 and S6. Thus, for the samples with 500 ≤ T_max.heat._ ≤ 800 °C, the change in saturation magnetization was primarily related to the change in particle size, which is consistent with previous studies of the Mn_x_Fe_3−x_O_4_ system [45,46,47]. Changes in saturation magnetization with the change in particle size can be explained by the existence of a non-magnetic layer [40,48]. This non-magnetic layer is associated with disordered magnetic dipoles on the surface of magnetic particles [49] due to the canting of the surface spins, high anisotropy layer, or loss of the long-range order in the surface layer [50,51]. Since the surface-to-volume ratio is inversely proportional to the particle size, the proportion of the surface layer increases with decreasing particle size, which leads to an increase in the number of disordered magnetic dipoles on the surface of nanoparticles and, consequently, to a decrease in saturation magnetization. An XRD analysis of the sample heated to 900 °C showed a significant increase in the content of antiferromagnetic MnO [52], which leads to a change in the magnetic properties of this sample.

As in the case of the as-synthesized sample, samples taken after high-temperature magnetic measurements have sufficiently low values of remanent magnetization and were fitted by the Langevin function (Figure 4c,d shows hysteresis loops fitted using the Langevin function for samples heated to 500 and 900 °C, respectively). The values of saturation magnetization ($M_{S}^{fit}$) and mean magnetic moment (*μ*) of all samples, obtained from Langevin fitting, are listed in Table 1. The change in μ values for samples S1–S4 correlates with the change in the crystallite size in these samples, which is in good agreement with the results obtained for the change in the mean magnetic moment in MnFe_2_O_4_ nanoparticles [53]. As follows from the TEM results, needle-like nanoparticles have a large particle size distribution, in the range from 30 to 130 nm; therefore, a decrease in the values of *μ* for samples S5–S7 may be related to the fact that, in these samples, some of the particles have a multi-domain structure, while another part is in a single-domain state.

From the magnetization curves, the average magnetic domain size was estimated for samples S2–S4 using Equation (3) [54]. However, such estimation is impossible for needle-like nanoparticles (samples S5–S7) since, to describe domains in nanoparticles of this type, at least two geometric parameters are required.

(3) $$d_{S}={\left[\frac{18k_{B}T}{\pi \rho M_{S}^{2}}{\left(\frac{dM}{dH}\right)}_{H\to 0}\right]}^{\frac{1}{3}}$$

where *Ms* is the saturation magnetization, dM/dH is the initial slope of the M–H curve near *H* = 0, *k_B_* is the Boltzmann constant, *ρ* is density, and *T* is temperature. The initial slopes of the samples near the origin were determined from the hysteresis curves by fitting the linear portion (H ~ 0) of the M–H curves. The calculated values of *d_S_* are summarized in Table 1. Assuming that the nanoparticles in samples S1–S4 have a spherical shape, the average number of magnetic domains per particle could be roughly estimated by dividing the particle volume by the average volume of the magnetic domain. Such an estimation provided the values of 5, 23, 15, and 3 magnetic domains per particle for samples S1–S4, respectively. Additionally, for samples S1 and S4, it was seen that magnetic domain sizes were smaller than crystallite sizes—although they are close to them—which can be explained by the presence of a non-magnetic layer on the surface of the nanoparticles. This result agreed with the above-discussed change in the saturation magnetization with a change in particle size due to the presence of disordered magnetic dipoles on the surface of magnetic nanoparticles.

## 4. Conclusions

The effect of the heating temperature on the structural and magnetic properties of Fe_1.1_Mn_1.9_O_4_ nanoparticles, synthesized using the combustion method, was studied. The morphological analysis of the as-synthesized sample showed that particles were almost uniform in size and had spherical or quasi-spherical shapes with an average particle size of ~24 nm. Furthermore, XRD analysis showed that the as-synthesized sample had a tetragonal structure, which can be associated with the influence of size effect on the redistribution of cations between the tetrahedral and octahedral sites. After high-temperature magnetic measurements, the samples were again subjected to structural and magnetic analyses. It has been found that, at 500 ≤ T_max.heat._ ≤ 600 °C, the samples retain their original tetragonal structure after cooling. However, a further increase in the maximum heating temperature leads to the structural transition becoming irreversible, and cooled samples retain the high-temperature cubic structure. The results of magnetic measurements showed that all samples demonstrated ferrimagnetic behavior at room temperature. The observed changes in magnetic parameters have been associated with changes in the size of nanoparticles and the formation of impurities during high-temperature measurements. 

## Figures and Tables

**Table 1:** Average crystallite size and magnetic parameters of the as-synthesized sample and samples after high-temperature magnetic measurements. Here, D is the average crystallite size, *M_S_* is the saturation magnetization, $M_{S}^{fit}$ is the value of saturation magnetization obtained from the best fitting of the curves using the Langevin function, *M_R_* is the remanent magnetization, *H_C_* is coercivity; *μ* is the mean magnetic moment, and *d_S_* is the average magnetic domain size.

Sample	S1	S2	S3	S4	S5	S6	S7
*D*, nm	20.5	33.1	29.5	19.4	-	-	-
*M_S_*, emu/g	20.9	38.8	33.9	15.2	19.5	32	21.3
$M_{S}^{fit}M_{S}^{fit}$, emu/g	21.5	36.2	32.1	15.1	20.3	32.2	21.5
*M_R_*, emu/g	2.3	6.4	6.1	1.9	1.6	2.7	1.14
*H_C_*, Oe	38.8	80.6	81.9	36.3	26.1	43.1	56.3
*μ*, μ_B_	20,644	28,551	26,794	18,448	14,934	14,055	9224
*d_S_*, nm	12.4	11.6	11.9	13.3	-	-	-

## Data Availability

The data presented in this study are available upon request by email: aleksandr.a.spivakov@gmail.com. The data are not publicly available due to they also form part of ongoing research.
